# Impact of Cardio-cerebrovascular Diseases on Enzalutamide and Abiraterone Treatment in Castration-resistant Prostate Cancer

**DOI:** 10.31662/jmaj.2026-0201

**Published:** 2026-07-31

**Authors:** Noriyasu Kawai, Kouji Izumi, Takahiro Yasui, Takashi Shima, Yuki Kato, Koji Mita, Takehiko Okamura, Shogo Inoue, Shuichi Tatarano, Nobumichi Tanaka, Manabu Kamiyama, Ippei Chikazawa, Seiji Hoshi, Kohei Hashimoto, Takashi Fukagai, Kazuyoshi Shigehara, Shizuko Takahara, Atsushi Mizokami

**Affiliations:** 1 Department of Nephro-urology, Nagoya City University Graduate School of Medical Sciences, Nagoya, Japan; 2 Department of Urology, Nagoya City University Midori Municipal Hospital, Nagoya, Japan; 3 Department of Integrative Cancer Therapy and Urology, Kanazawa University Graduate School of Medical Science, Kanazawa, Japan; 4 Department of Urology, Toyama Prefectural Central Hospital, Toyama, Japan; 5 Department of Urology, Fukui-ken Saiseikai Hospital, Fukui, Japan; 6 Department of Urology, Hiroshima City North Medical Center Asa Citizens Hospital, 1Hiroshima, Japan; 7 Department of Urology, Anjo Kosei Hospital, Anjo, Japan; 8 Department of Urology, Kobatake Hospital, Fukuyama, Japan; 9 Department of Urology, Graduate School of Medical and Dental Sciences, Kagoshima University, Kagoshima, Japan; 10 Department of Urology, Nara Medical University, Kashihara, Japan; 11 Department of Urology, University of Yamanashi Graduate School of Medical Sciences, Chuo, Japan; 12 Department of Urology, Kanazawa Medical University, Kahoku, Japan; 13 Department of Urology, Fukushima Medical University School of Medicine, Fukushima, Japan; 14 Department of Urology, School of Medicine, Sapporo Medical University, Sapporo, Japan; 15 Department of Urology, Showa University School of Medicine, Tokyo, Japan; 16 Department of Urology, Ishikawa Prefectural Central Hospital, Kanazawa, Japan; 17 Innovative Clinical Research Center, Kanazawa University, Kanazawa, Japan; 18 Medical Research Support Center, University of Fukui Hospital, Fukui, Japan

**Keywords:** androgen receptor signaling inhibitor, cardiovascular diseases, castration-resistant, cerebrovascular diseases, survival outcomes

## Abstract

**Introduction:**

The ENABLE study demonstrated comparable survival benefits between enzalutamide (ENZ) and abiraterone plus prednisolone (ABI) in castration-resistant prostate cancer (CRPC). Although the association between androgen receptor signaling inhibition and cardio-cerebrovascular diseases (CCVDs) has long been discussed, definitive evidence remains limited. We evaluated the efficacy of these agents in patients with CRPC who have CCVDs.

**Methods:**

This investigator-initiated, multicenter, randomized controlled trial allowed enrollment of patients with prior or concurrent CCVDs. Prespecified end points included time to prostate-specific antigen (PSA) progression (TTPP), radiographic progression-free survival (rPFS), overall survival (OS), prostate cancer–specific survival (PCSS), and safety. Outcomes were compared between patients with and without CCVDs (CCVD− vs CCVD+) and between treatment arms within each group.

**Results:**

The CCVD− group included 142 patients (75 ENZ and 67 ABI), and the CCVD+ group included 36 patients (14 ENZ and 22 ABI). The most frequent CCVD was cerebral infarction (n = 11, 31%). Survival outcomes did not differ significantly between patients in the CCVD− group and those in the CCVD+ group (median TTPP 15.2 vs 21.2; p = 0.8001; rPFS 18.1 vs 9.4; p = 0.0971; OS 37.4 vs 30.5; p = 0.1793; PCSS 46.8 vs 32.1; p = 0.6348). Within both groups, ENZ and ABI showed no significant differences in TTPP, rPFS, OS, or PCSS. Adverse event rates were also comparable across CCVD groups and treatment arms.

**Conclusions:**

Patients with CCVDs showed no significant differences in survival or safety compared with those without CCVDs, and ENZ and ABI produced similar outcomes in both groups. These findings indicate that both agents remain feasible options irrespective of CCVD history.

**Public trials registry:**

The trial was registered with the University Hospital Medical Information Network (UMIN) Center under the identifier UMIN000015529 on November 1, 2014.

## Introduction

Prostate cancer is the most frequently diagnosed malignancy and the second leading cause of cancer-related mortality among American men ^(1)^. Its incidence has also been steadily increasing in Japan ^(2)^. For patients with advanced disease, androgen deprivation therapy (ADT), the basic approach to suppress androgen receptor (AR) signaling, remains the cornerstone of treatment. However, most prostate cancers inevitably progress to castration-resistant prostate cancer (CRPC) within a few years of initiating ADT ^(3), (4), (5), (6), (7)^. Recent evidence indicates that AR signaling inhibitors (ARSIs) prolong radiographic progression-free survival (rPFS) and improve overall survival (OS) in patients with CRPC ^(8), (9)^. Enzalutamide (ENZ) exerts its antitumor activity by directly binding to the ligand-binding domain of the AR ^(9)^, whereas abiraterone plus prednisolone (ABI) suppresses androgen biosynthesis through inhibition of CYP17A1, a key enzyme in steroidogenesis ^(10)^. Given that ARSIs converge on a common signaling pathway, survival outcomes reported for ENZ and ABI have generally been comparable ^(11), (12)^. However, the association between AR signaling inhibition and cardio-cerebrovascular diseases (CCVDs) has long been debated. Potential disadvantages of ABI with respect to CCVD risk have drawn attention, although definitive evidence remains lacking. To address this issue, we evaluated the efficacy of these agents in patients with CRPC who had a history of CCVDs as a subanalysis of the ENABLE study for PCa.

## Materials and Methods

### Study design

The ENABLE study for PCa was a multicenter, investigator-initiated randomized controlled trial that compared the therapeutic efficacy of ENZ and ABI in patients with CRPC. Informed consent was obtained from all participants. Details regarding the inclusion criteria and treatment procedures have been reported elsewhere ^(13), (14), (15), (16), (17)^. Briefly, participants were centrally randomized in a 1:1 allocation by the data center of the Innovative Clinical Research Center at Kanazawa University to receive either ENZ (four 40 mg tablets once daily; 160 mg/day) or ABI (four 250 mg tablets once daily; 1,000 mg/day) in combination with prednisolone (5 mg twice daily; 10 mg/day). Dose reductions at treatment initiation and the choice of subsequent therapies after study discontinuation were left to the discretion of the treating physicians. The protocol also permitted the enrollment of patients with a history of CCVDs, whether previous or ongoing, at the discretion of the attending physician. One participating site requested the withdrawal of its data after study completion; therefore, the data of six patients from that site were excluded from the originally collected dataset.

### Definition of end points

Time to prostate-specific antigen (PSA) progression (TTPP) was assessed based on the criteria established by the Prostate Cancer Working Group 2 (PCWG2) ^(4)^. In patients who exhibited a PSA decline at week 13, the date of PSA progression was defined as the first occurrence of a ≥25% PSA increase and an absolute rise of ≥2 ng/mL above the nadir, confirmed by a second consecutive PSA measurement obtained at least 3 weeks later. For patients without a PSA decline at week 13, progression was defined as a ≥25% and ≥2 ng/mL PSA increase above the baseline, similarly confirmed by a second value after a minimum interval of 3 weeks. In cases where treatment was discontinued before week 13 without a PSA decrease, the date of discontinuation was considered as the PSA progression date; however, this definition does not follow the PCWG2 criteria. TTPP was calculated as the interval from randomization to the first confirmed PSA progression event.

The other end points are defined as follows: PSA response rate, defined as a ≥50% decline in PSA level from baseline; rPFS, assessed according to the Response Evaluation Criteria in Solid Tumors version 1.1 for soft-tissue lesions (evaluated by computed tomography or magnetic resonance imaging) and PCWG2 criteria for bone metastases (evaluated by bone scintigraphy); OS, defined as the time from randomization to death from any cause; prostate cancer–specific survival (PCSS), defined as the time from randomization to death due to prostate cancer; docetaxel (DTX)-free survival (DFS), defined as the time from randomization to the initiation of DTX treatment; performance status (PS) progression-free survival (PSPFS), defined as the time from randomization to deterioration of PS as evaluated using The Eastern Cooperative Oncology Group PS scale (https://ecog-acrin.org/resources/ecog-performance-status/).

### Safety assessment

Safety was assessed according to the frequency and severity of adverse events (AEs) using the Common Terminology Criteria for Adverse Events, version 4.0 (http://evs.nci.nih.gov/ftp1/CTCAE/About.html).

### End point analysis

First, end points in the overall population, including both ENZ and ABI arms, were analyzed according to the presence (CCVD+) or absence (CCVD−) of CCVDs history. This initial comparison was intended to clarify whether a prior or coexisting history of CCVDs influenced the overall efficacy and safety outcomes observed in the trial population. Next, end points in the CCVD− and CCVD+ groups were analyzed separately by treatment arm (ENZ or ABI), allowing us to determine whether the impact of CCVDs differed between the two ARSIs. Information on CCVDs was recorded at the time of patient enrollment.

### Statistical analyses

Detailed information on the statistical analyses is provided in our previous report ^(13)^. Baseline characteristics were first compared between patients in the CCVD− and CCVD+ groups, and then between patients in the ENZ and ABI arms within each CCVD− and CCVD+ subgroup. Continuous variables were analyzed using an unpaired t-test. Categorical variables, including PS, presence or absence of regional lymph node metastasis, and presence or absence of distant metastasis, were compared using Fisher’s exact test. Gleason scores were analyzed using the chi-square test for trend. Survival curves were estimated using the Kaplan–Meier method, and differences between CCVD groups or treatment arms were assessed with the log-rank test. PSA response rates were compared using Fisher’s exact test. All patients were evaluated for ARSI-related toxicity, and the frequencies of any AEs and grade ≥3 AEs were compared between groups using Fisher’s exact test. All statistical analyses were two-sided, and p value <0.05 was considered statistically significant.

## Results

From February 2015 to July 2019, a total of 197 patients were enrolled, of whom 178 were randomly assigned to either the ENZ or ABI treatment arm (89 patients each) and received the study drugs. Of these, 142 patients were in the CCVD− group and 36 in the CCVD+ group. Table 1 summarizes the baseline characteristics of the patients, with no significant differences observed between the CCVD− and CCVD+ groups.

**Table 1.** Baseline Characteristics at Randomization.

		CCVD−		p Value	CCVD+		p Value	p Value CCVD− vs CCVD+
		ENZ	ABI		ENZ	ABI		
n		75	67		14	22		
Age (years)		74 (46-88)	77 (53-92)	0.1113	79.5 (65-88)	77 (65-85)	0.4199	0.1758
Performance status				>0.9999			0.4672	0.2781
	0	59 (79)	52 (78)		11 (79)	14 (64)		
	1	16 (21)	15 (22)		3 (21)	8 (36)		
Gleason score				0.0804			0.5485	0.4043
	5-7	8 (11)	13 (19)		3 (21)	3 (14)		
	8	14 (19)	16 (24)		3 (21)	5 (23)		
	9	43 (57)	28 (42)		8 (57)	12 (55)		
	10	9 (12)	7 (10)		0	1 (5)		
	Not available	1 (1)	3 (4)		0	1 (5)		
Local treatment*				>0.9999			0.0924	0.1481
	Prostatectomy	3 (4)	4 (6)		2 (14)	1 (5)		
	Irradiation†	16 (21)	13 (19)		6 (42)	5 (23)		
	None	55 (73)	48 (72)		6 (42)	16 (73)		
Regional lymph node metastasis				0.6017			0.4715	>0.9999
	Yes	28 (37)	22 (33)		6 (43)	6 (27)		
	No	47 (63)	45 (67)		8 (57)	16 (73)		
Distant metastasis				0.1587			0.4315	0.825
	Bone	52 (69)	47 (70)		8 (57)	13 (59)		
	Lymph node	17 (23)	7 (10)		6 (43)	6 (27)		
	Lung	5 (7)	6 (9)		2 (14)	1 (5)		
	Liver	1 (1)	1 (1)		0	0		
	Others	0	1 (1)		1 (7)	0		
	No	13 (17)	19 (28)		2 (14)	7 (32)		
No. of previous systemic therapy‡		2 (1-5)	3 (1-5)	0.4268	3 (1-4)	2 (1-5)	0.5164	0.9788
Prostate-specific antigen (ng/mL)								
	At diagnosis§	114 (1.8-5400)	102 (0.9-3888)	0.4939	64.2 (9.0-2923)	112 (12.7-6010)	0.7966	0.2178
	At nadir before registration||	0.3 (0.003-185)	0.4 (0.001-28.3)	0.0565	0.1 (0.003-2.3)	0.829 (0.004-68.7)	0.2035	0.5468
	At registration	6.8 (2.0-327)	10.8 (2.0-479)	0.264	7.1 (2.1-82.2)	12.2 (2.4-549)	0.2891	0.688
Time from diagnosis of prostate cancer to randomization (months)¶		29.0 (3.0-197)	25.8 (4.5-190)	0.609	54.8 (9.8-142)	23.8 (7.6-111)	0.023	0.6803
Time from castration resistance to randomization (months)**		1.4 (0.0-122)	1.3 (0.0-56.2)	0.263	1.6 (0.0-23.3)	1.2 (0.0-37.4)	0.543	0.9761

Data are median (range), n (%). ABI: abiraterone plus prednisolone; CCVD: cardio-cerebrovascular disease; ENZ: enzalutamide. *Data of 3 in the CCVD− group are not available. †Including high and low dose rate brachytherapy, and external beam radiation therapy for primary site. ‡Medical or surgical castration is counted as 1, and data of 3 in CCVD− are not available. §Data of 1 in the CCVD+ group is not available. ||Data of 5 in the CCVD− group and 2 in the CCVD+ group are not available. ¶Data of 2 in the CCVD− group and 1 in the CCVD+ group are not available. **Data of 7 in the CCVD− group and 6 in the CCVD+ group are not available.

Median TTPP and rPFS in the CCVD− and CCVD+ groups were 15.2 and 21.2 months, and 18.1 and 9.4 months, respectively (p = 0.8001 and p = 0.0971; Figure 1A and B). Median OS and PCSS in the CCVD− and CCVD+ groups were 37.4 and 30.5 months, and 46.8 and 32.1 months, respectively (p = 0.1793 and p = 0.6348; Figure 1C and D). Median DFS and PSPFS in the CCVD− and CCVD+ groups were 19.0 and 16.0 months, and 35.5 and 27.7 months, respectively (p = 0.3721 and p = 0.1824; Supplementary Figure 1A and B). There was no significant difference in PSA response rate between the CCVD− and CCVD+ groups (64% and 64%, respectively; p ≥ 0.9999; Supplementary Figure 1C).

**Figure 1.**
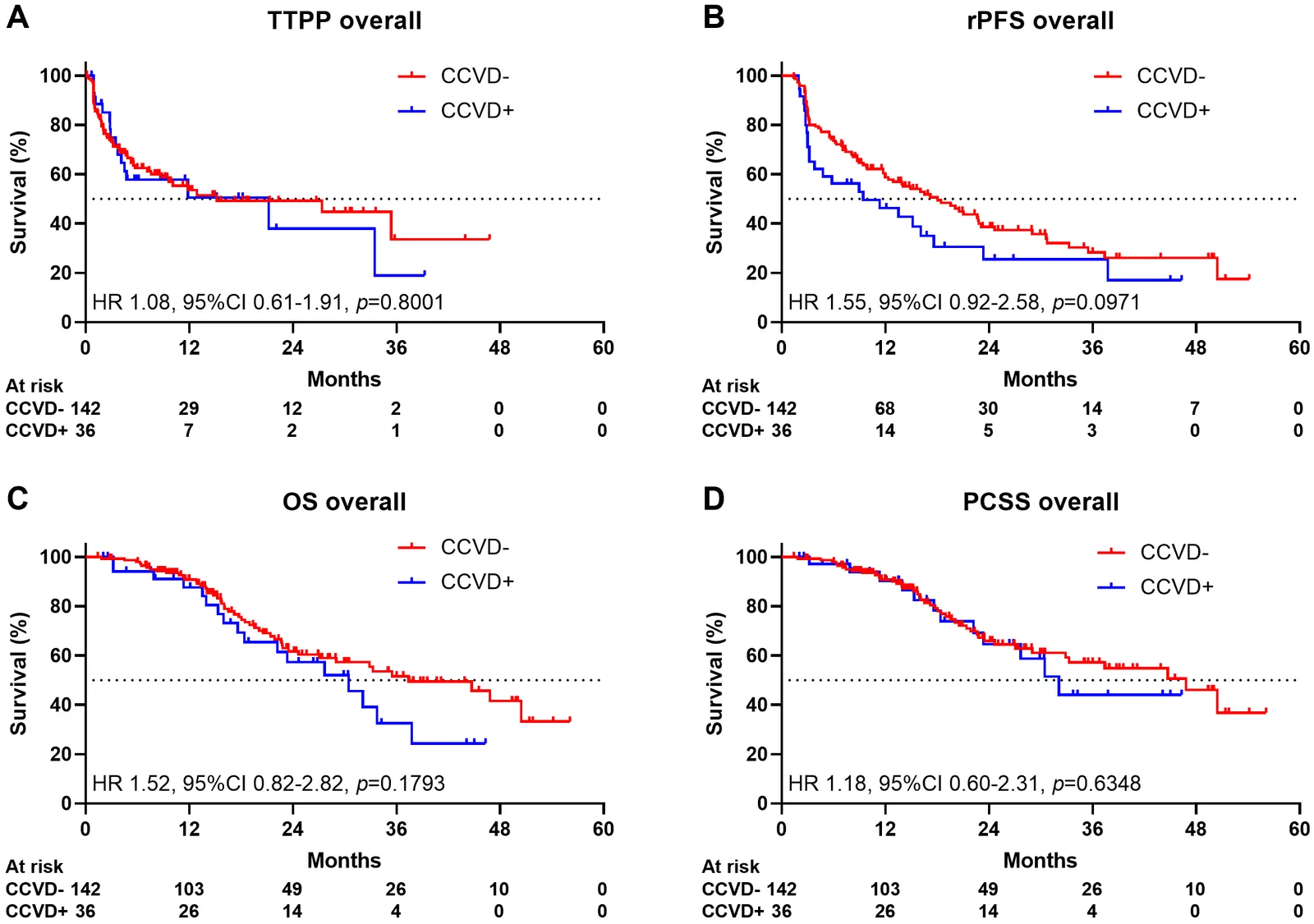
Comparisons of survival end points according to the presence (CCVD+) or absence (CCVD−) of history of cardio-cerebrovascular diseases (CCVDs) in the overall cohort. A, time to PSA progression (TTPP). B, radiographic progression-free survival (rPFS). C, overall survival (OS). D, prostate cancer–specific survival (PCSS). CI: confidence interval; HR: hazard ratio; PSA: prostate-specific antigen.

As shown in Table 1, no significant differences were observed between the ENZ and ABI arms in the CCVD− group. In the analysis of the CCVD− group, no significant differences were observed in any survival outcomes between the ENZ and ABI arms. Median TTPP and rPFS in the ENZ and ABI arms were 15.2 and 10.1 months, and 18.1 and 20.5 months, respectively (p = 0.3968 and p = 0.5578; Figure 2A and B). Median OS and PCSS in the ENZ and ABI arms were 46.8 and 37.4 months, and 46.8 and 44.7 months, respectively (p = 0.7433 and p = 0.4358; Figure 2C and D). Median DFS and PSPFS in the ENZ and ABI arms were 19.3 and 16.5 months, and 46.8 and 35.5 months, respectively (p = 0.9330 and p = 0.9727; Supplementary Figure 2A and B). There was a significant difference in PSA response rate between the ENZ and ABI arms (73% and 54%, respectively; p = 0.0221; Supplementary Figure 2C).

**Figure 2.**
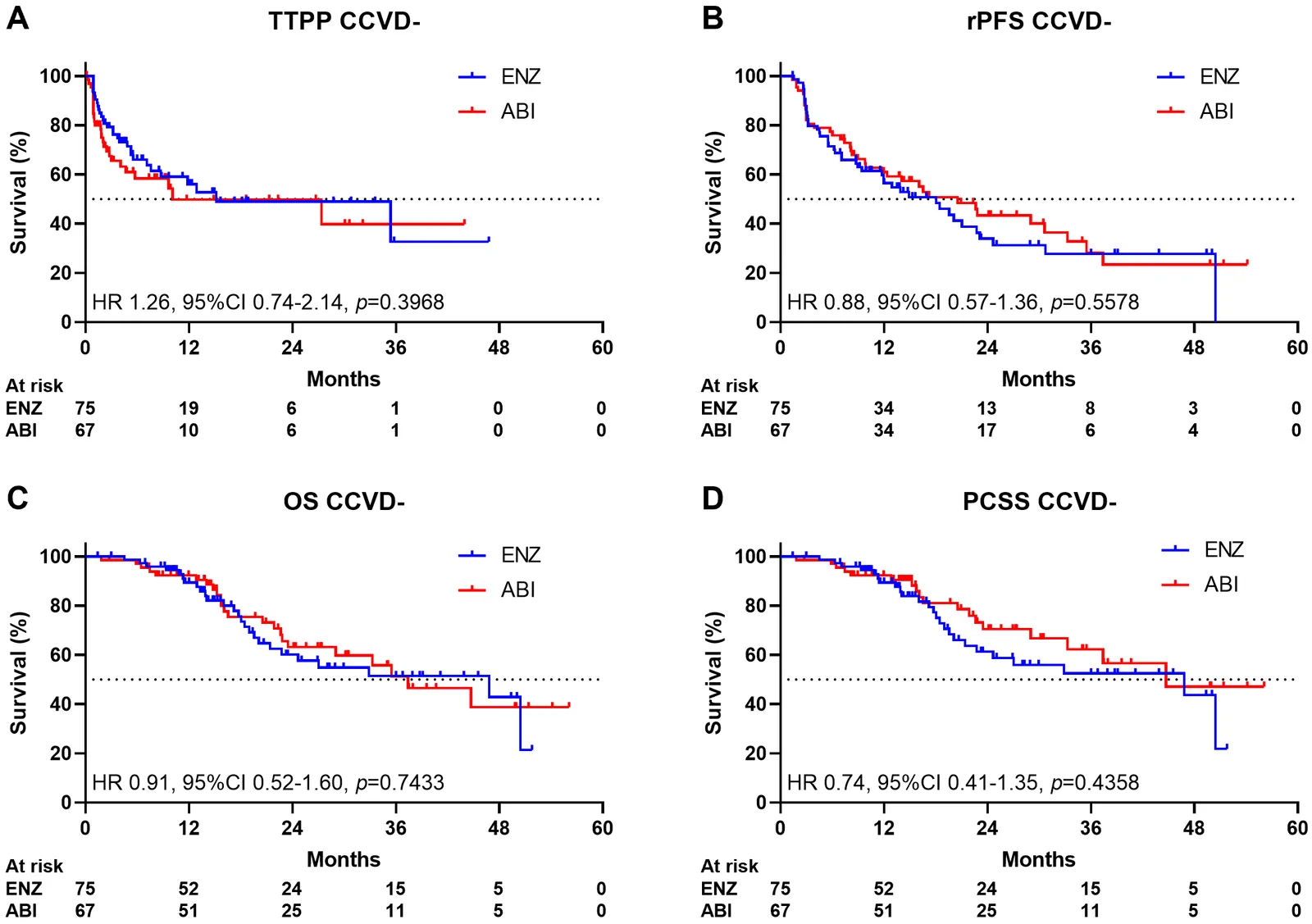
Comparisons of survival end points between the enzalutamide (ENZ) and abiraterone plus prednisolone (ABI) arms in the absence (CCVD−) of cardio-cerebrovascular diseases (CCVDs). A, time to PSA progression (TTPP). B, radiographic progression-free survival (rPFS). C, overall survival (OS). D, prostate cancer–specific survival (PCSS). CI: confidence interval; HR: hazard ratio; PSA: prostate-specific antigen.

In the analysis of the CCVD+ group, no significant differences in baseline characteristics were observed between the ENZ and ABI arms, except for time from diagnosis of prostate cancer to randomization (Table 1), and no significant differences were found in any survival outcomes. Median TTPP and rPFS in the ENZ and ABI arms were 21.2 and 11.9 months, and 17.6 and 4.3 months, respectively (p = 0.4891 and p = 0.1252; Figure 3A and B). Median OS and PCSS in the ENZ and ABI arms were 22.2 and 30.5 months, and undefined and 30.5 months, respectively (p = 0.5610 and p = 0.5812; Figure 3C and D). Median DFS and PSPFS in the ENZ and ABI arms were 16.0 and 18.4 months, and 23.3 and 30.5 months, respectively (p = 0.5741 and p = 0.6398; Supplementary Figure 3A and B). There was no significant difference in PSA response rate between the ENZ and ABI arms (71% and 59%, respectively; p = 0.5013; Supplementary Figure 3C).

**Figure 3.**
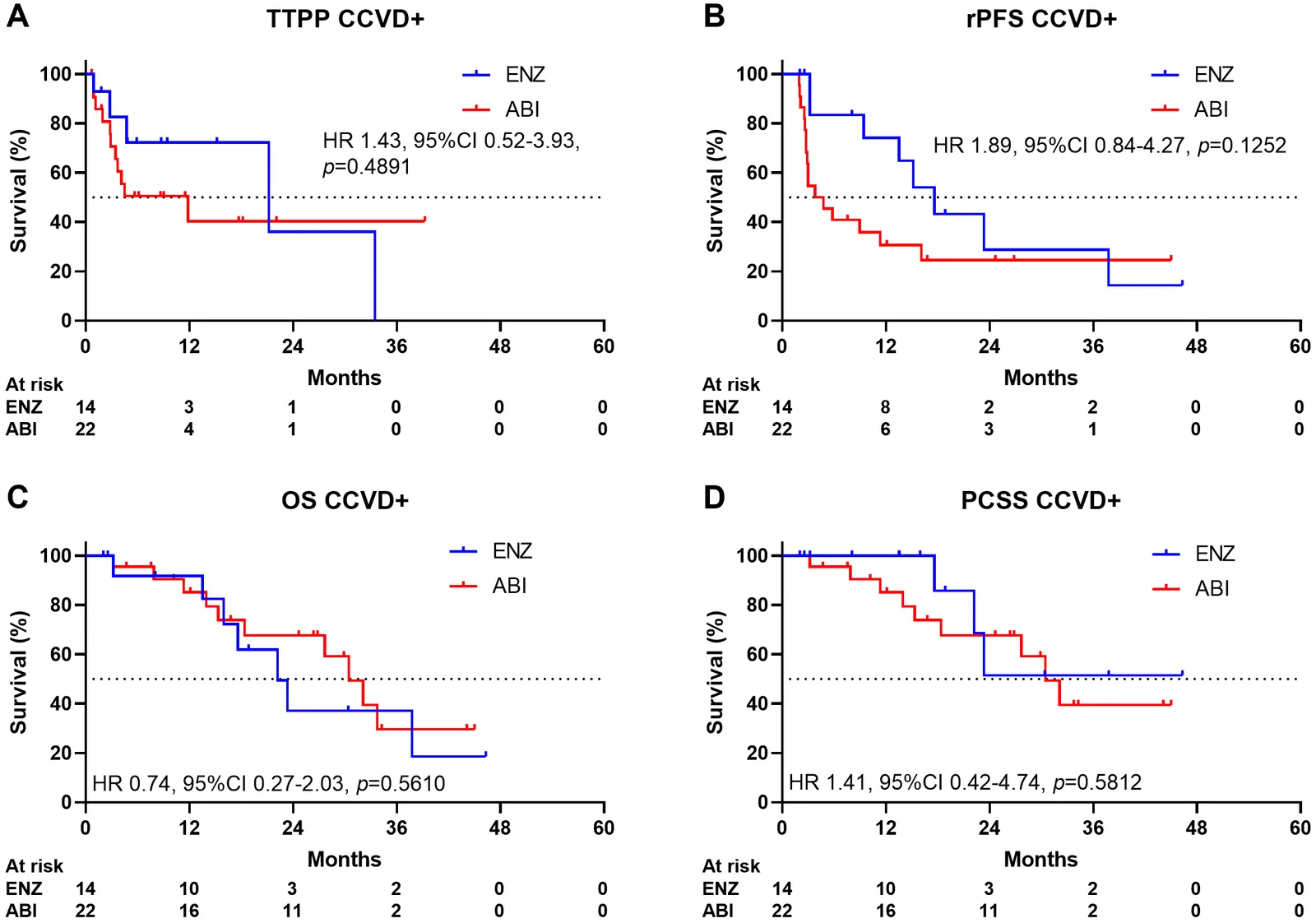
Comparisons of survival end points between the enzalutamide (ENZ) and abiraterone plus prednisolone (ABI) arms in the presence (CCVD]+) of cardio-cerebrovascular diseases (CCVDs). A, time to PSA progression (TTPP). B, radiographic progression-free survival (rPFS). C, overall survival (OS). D, prostate cancer–specific survival (PCSS). CI: confidence interval; HR: hazard ratio; PSA: prostate-specific antigen.

Table 2 summarizes the types and number of past or concurrent CCVDs in the CCVD+ group. The most frequent CCVD was cerebral infarction (n = 11, 31%), followed by atrial fibrillation, myocardial infarction, and angina pectoris (n = 7, 19% each). In the CCVD+ group, three patients died from CCVDs (heart failure, congestive heart failure, and rupture of thoracic aortic aneurysm), whereas one died from CCVD (heart failure) in the CCVD− group.

**Table 2.** Types and Number of Past or Concurrent CCVDs.

		ENZ	ABI
n		14	22
Types of CCVDs			
	Atrial fibrillation	2 (14)	5 (23)
	Myocardial infarction	4 (29)	3 (14)
	Angina pectoris	2 (14)	5 (23)
	Ischemic heart disease	1 (7)	0
	Heart failure	1 (7)	2 (9)
	Congestive heart failure	0	1 (5)
	Mitral regurgitation	0	1 (5)
	Pulmonary valve stenosis	0	1 (5)
	Hypertrophic cardiomyopathy	0	1 (5)
	Ventricular septal defect	0	1 (5)
	Ventricular aneurysm	1 (7)	0
	Cerebral infarction	5 (36)	6 (27)
	Intracerebral hemorrhage	2 (14)	2 (9)
	Chronic subdural hematoma	0	1 (5)
	Internal carotid artery stenosis	1 (7)	0
	Vertebrobasilar insufficiency	1 (7)	0
	Congenital cerebral aneurysm	0	1 (5)
	Arteriosclerosis obliterans	1 (7)	1 (5)
	Venous thrombosis	0	1 (5)
Number of CCVDs per patient			
	4	1 (7)	1 (5)
	3	1 (7)	1 (5)
	2	2 (14)	5 (23)
	1	10 (71)	15 (68)

Data are n (%). ABI: abiraterone plus prednisolone; CCVD: cardio-cerebrovascular disease; ENZ: enzalutamide.

Table 3 summarizes AEs of patients stratified by treatment arm. There were no significant differences in the incidences of any grade or grade ≥3 AEs between the CCVD− and CCVD+ groups (p > 0.9999 and p > 0.9999), between the ENZ and ABI arms of the CCVD− group (p = 0.8586 and p = 0.1789), or between the ENZ and ABI arms of the CCVD+ group (p = 0.4715 and p = 0.3705). With regard to CCVD-related AEs, no significant differences were observed in the incidence of either any-grade or grade ≥3 AEs between the CCVD− and CCVD+ groups (p = 0.7683 and p > 0.9999). Similarly, no significant differences were found between the ENZ and ABI arms within the CCVD− group (p = 0.2876 and p = 0.1884), or within the CCVD+ group (p = 0.5193 and p = 0.3889).

**Table 3.** Adverse Events.

			CCVD−		p Value	CCVD+		p Value	p Value CCVD− vs CCVD+
			ENZ	ABI		ENZ	ABI		
n			75	67		14	22		
Any grade			51 (68)	44 (66)	0.8586	8 (57)	16 (73)	0.4715	>0.9999
	CCVD-related		6 (8)	10 (15)	0.2876	1 (7)	2 (9)	0.5193	0.7683
		Hypertension	3	3		0	2		
		Edema	1	4		0	0		
		Atrial fibrillation	1	0		0	0		
		ST elevation in ECG	1	0		0	0		
Grade ≥3			9 (12)	14 (21)	0.1789	1 (7)	5 (23)	0.3705	>0.9999
	CCVD-related		1 (1)	4 (6)	0.1884	1 (7)	0	0.3889	>0.9999
		Hypertension	0	2		0	0		
		Edema	0	1		0	0		
		Acute myocardial infarction	0	1		0	0		
		Arrhythmia	1	0		0	0		
		Rupture of aortic aneurysm	0	0		1	0		

Data are n (%). Event counts represent the total number of events. ABI: abiraterone plus prednisolone; CCVD: cardio-cerebrovascular disease; ECG: electrocardiogram; ENZ: enzalutamide.

## Discussion

Previously, we reported that the intention-to-treat analysis of the ENABLE study showed no significant differences in TTPP or other survival end points between the ENZ and ABI arms in patients with CRPC ^(13)^. In this study, we evaluated whether a history of CCVDs modify the treatment effects of ENZ and ABI. Randomized controlled trials often exclude patients with a history of CCVDs because these conditions introduce competing mortality risks that can obscure the true effects of the investigational cancer therapy on survival end points such as OS and PFS. Moreover, CCVDs and their treatments can independently increase the likelihood of AEs during cancer therapy, making it difficult to distinguish toxicities caused by the study drug from those attributable to underlying CCVDs. Indeed, 34% of pivotal cancer clinical trials excluded patients with cardiovascular diseases ^(18)^. A systematic review and meta-analysis reported that ARSIs were associated with an increased risk of all-grade CCVD events, including cerebrovascular events and CCVD-related mortality ^(19)^. However, there is limited evidence regarding whether a history of CCVDs modifies the treatment effects of ENZ or ABI.

Our data clearly showed that, in the overall cohort, the CCVD+ group did not adversely affect any prostate cancer–related survival outcomes. This suggests that ARSIs exert comparable efficacy in patients in both CCVD+ and CCVD− groups.

In the analysis of the CCVD− group, no significant differences were observed in any survival end points between the ENZ and ABI arms; however, a significant difference was noted in the PSA response rate. These findings were consistent with those of the intention-to-treat analysis, suggesting that patients with CCVD− largely reflect the overall cohort of the ENABLE study for PCa. In the analysis of the CCVD+ group, the types of past or concurrent CCVDs were generally comparable between the ENZ and ABI arms. The most frequent CCVD was cerebral infarction, followed by atrial fibrillation, myocardial infarction, and angina pectoris. The distribution of the number of CCVDs per patient was also similar. As in the CCVD− group, no significant differences in any survival end points were observed between the ENZ and ABI arms, indicating that both ENZ and ABI can exert their oncological efficacy in patients with CCVDs. Importantly, no significant differences were observed in the incidence of any-grade or grade ≥3 AEs between the CCVD− and CCVD+ groups, between the ENZ and ABI arms within the CCVD− group, or between the ENZ and ABI arms within the CCVD+ group. These findings suggest that CCVDs do not impose additional negative effects on patients with CRPC who are receiving ARSIs.

However, systematic reviews have demonstrated that ARSIs are associated with an increased risk of cardiovascular deaths and events, including cardiac disorders, heart failure, atrial fibrillation, and acute myocardial infarction ^(20), (21), (22)^. An elevated risk of cognitive impairment and cerebrovascular events with ARSI use have also been reported ^(23)^. Among ARSIs, ABI has been particularly highlighted as a CCVD risk factor in patients with prostate cancer. A German study showed that ENZ was associated with a significantly lower risk of cardiovascular events compared with ABI in CRPC ^(24)^. An increased risk of cardiovascular-related hospitalization has also been reported in patients treated with ENZ and ABI, with ABI showing a higher risk than ENZ (9.8 vs. 7.1 events per 100 person-years, respectively) ^(25)^. In contrast, our findings were not consistent with these previously reported data. A history of CCVDs may not necessarily increase the likelihood of additional CCVD events during ARSI treatment, possibly because appropriate management of pre-existing CCVDs may mitigate further risk. Regarding OS, the CCVD+ group exhibited an increase in events after 24 months, suggesting that a longer follow-up period may better differentiate outcomes between the two groups.

This study has several limitations. First, the sample size was small, with only 36 patients in the CCVD+ group, who were further divided into the ENZ and ABI arms. This small sample size may have obscured potential differences between patients in the CCVD+ and CCVD− groups, including possible treatment-related deterioration of CCVD conditions. As a result, statistical analyses may have lacked sufficient power to detect clinically meaningful differences. Second, the study population consisted exclusively of Japanese patients, and individuals from other racial backgrounds were not represented. Third, the status of patients in the CCVD+ group was not clearly characterized; it remained unclear whether their CCVDs were historical or concurrent, active or stable, and whether they were receiving pharmacologic therapy, particularly antiplatelet agents or anticoagulants, or had undergone cardiovascular procedures, as well as whether such treatments were continued or discontinued during the study period. For example, the severity of CCVDs is known to increase cancer-related mortality, and experimental data from murine models have shown that circulating factors associated with heart failure can promote tumor growth ^(26), (27)^. In addition, ARSIs can significantly affect the metabolism of direct oral anticoagulants ^(28)^. These unmeasured factors may therefore have influenced both survival and safety outcomes in our cohort. In addition, potential biases related to dose selection, treatment discontinuation, and interpretation of treatment-course data may have arisen because the study was conducted in an open-label design. Finally, except for certain predefined conditions, patients with comorbidities, including other cancers, were included. These limitations may weaken the strength of the evidence regarding oncological outcomes and safety analyses.

In conclusion, this study found that patients in the CCVD+ group had survival outcomes and safety profiles comparable to those in the CCVD−group, and no significant differences were observed between the ENZ and ABI arms in either group. These results indicate that both ENZ and ABI may be reasonable treatment options irrespective of CCVD history. Nevertheless, considering the methodological constraints and limited clinical information available in our dataset, the results warrant cautious interpretation.

## Article Information

### Author Contributions

Conceptualization, Kouji Izumi; methodology, Kouji Izumi; validation, Noriyasu Kawai, Kouji Izumi, and Atsushi Mizokami; formal analysis, Kouji Izumi; investigation, Seiji Hoshi, Kouji Izumi, Takahiro Yasui, Takashi Shima, Yuki Kato, Koji Mita, Takehiko Okamura, Shogo Inoue, Shuichi Tatarano, Nobumichi Tanaka, Manabu Kamiyama, Ippei Chikazawa, Noriyasu Kawai, Kohei Hashimoto, Takashi Fukagai, Kazuyoshi Shigehara, and Atsushi Mizokami; resources, Kouji Izumi; data curation, Shuichi Tatarano; writing—original draft preparation, Noriyasu Kawai and Kouji Izumi; writing—review and editing, Kouji Izumi; visualization, Kouji Izumi; supervision, Atsushi Mizokami; project administration, Kouji Izumi; and funding acquisition, Kouji Izumi. All authors have read and approved the final version of the manuscript.

### Conflicts of Interest

Nobumichi Tanaka holds a position in an endowed chair supported by Nihon Medi-Physics Co. The other authors state that there are no conflicts of interest.

### Ethics Approval

The study was approved by the Medical Ethics Committee of Kanazawa University, Kanazawa, Japan, under reference number 2014-031.

### Data Availability Statement

The data are not publicly available due to privacy or ethical restrictions.

## Supplement

Supplementary Material
