# Supplementary material for: Impact of Cardio-cerebrovascular Diseases on Enzalutamide and Abiraterone Treatment in Castration-resistant Prostate Cancer
Source: JMA J. 2026 Jul 31;9(5):1350–9. doi: 10.31662/jmaj.2026-0201 (PMC13639697; doi:10.31662/jmaj.2026-0201)

Supplementary Figure 1. Comparisons of other survival end points and prostate-specific antigen (PSA) response between patients with (CCVD+) or without (CCVD−) a history of cardio-cerebrovascular disease (CCVD) in the overall cohort. A, docetaxel-free survival (DFS). B, performance status progression-free survival (PSPFS). C, waterfall plot of PSA response, defined as a ≥50% decline from baseline. Data of 2 in the CCVD− and 1 in the CCVD+ are not available in the analysis of PSPFS. Data of 3 in the CCVD− are not available in the analysis of PSA response. CI: confidence interval; HR: hazard ratio.

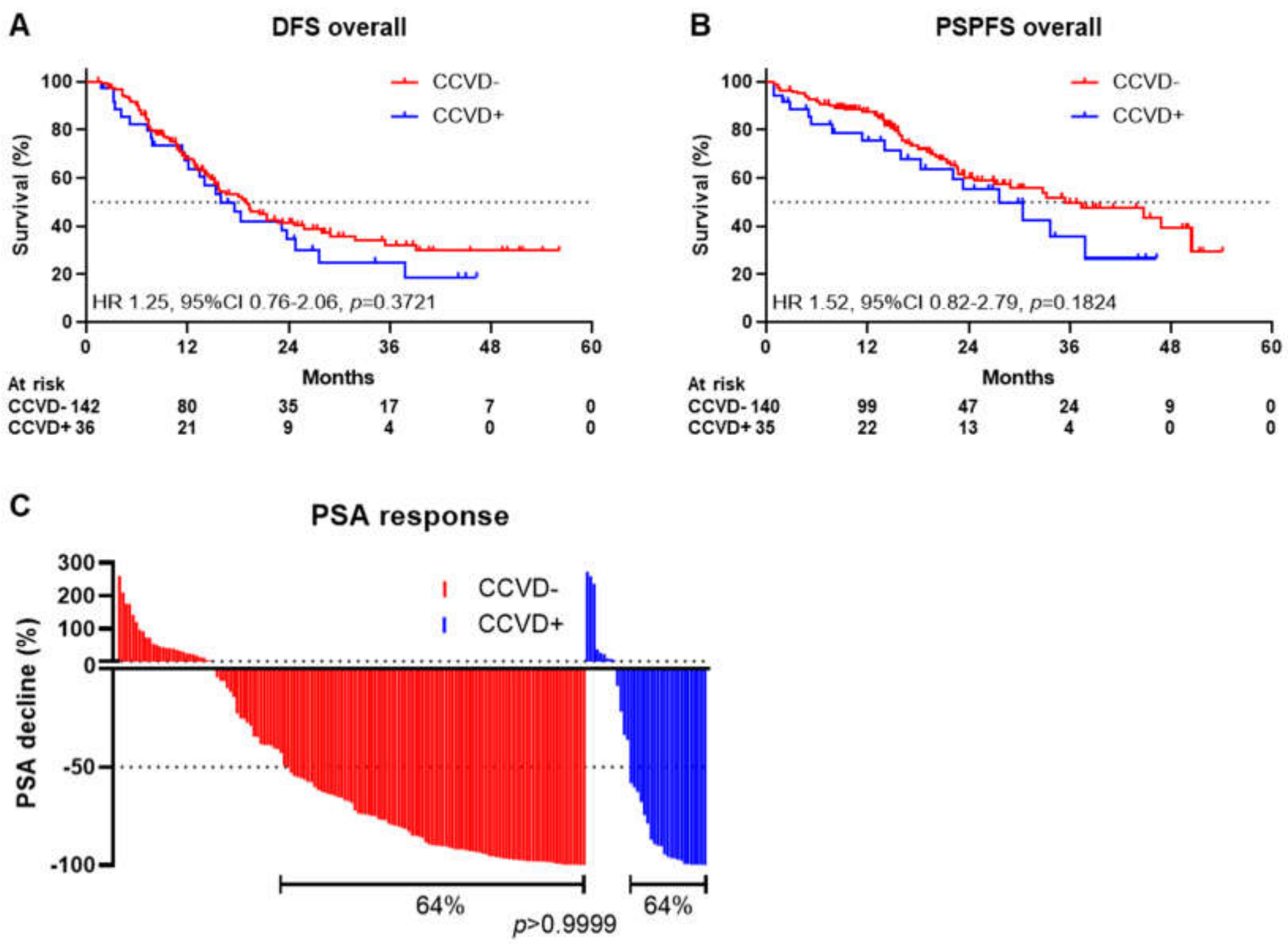

Supplementary Figure 2. Comparisons of other survival end points and prostate-specific antigen (PSA) response between the enzalutamide (ENZ) and abiraterone plus prednisolone (ABI) arms in the absence of history of cardio-cerebrovascular disease (CCVD−) group. A, docetaxel-free survival (DFS). B, performance status progression-free survival (PSPFS). C, waterfall plot of PSA response, defined as a ≥50% decline from baseline. Data of 2 in the ABI arm are not available in the analysis of PSPFS. Data of 1 in the ENZ arm and 2 in the ABI arm are not available in the analysis of PSA response. CI: confidence interval; HR: hazard ratio.

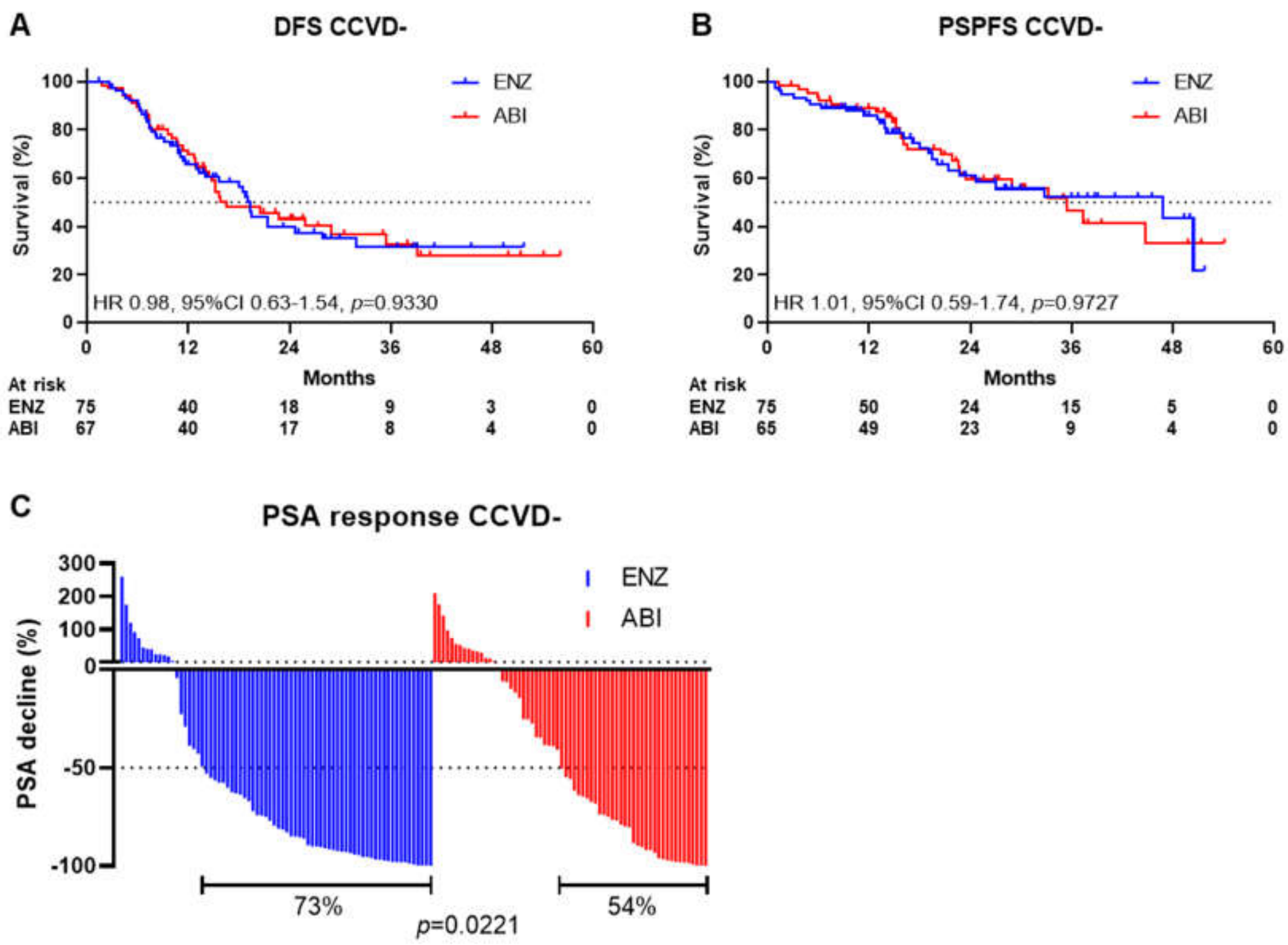

Supplementary Figure 3. Comparisons of other survival end points and prostate-specific antigen (PSA) response between the enzalutamide (ENZ) and abiraterone plus prednisolone (ABI) arms in the presence of history of cardio-cerebrovascular disease (CCVD+) group. A, docetaxel-free survival (DFS). B, performance status progression-free survival (PSPFS). C, waterfall plot of PSA response, defined as a ≥50% decline from baseline. Data of 1 in the ABI arm are not available in the analysis of PSPFS. CI: confidence interval; HR: hazard ratio.

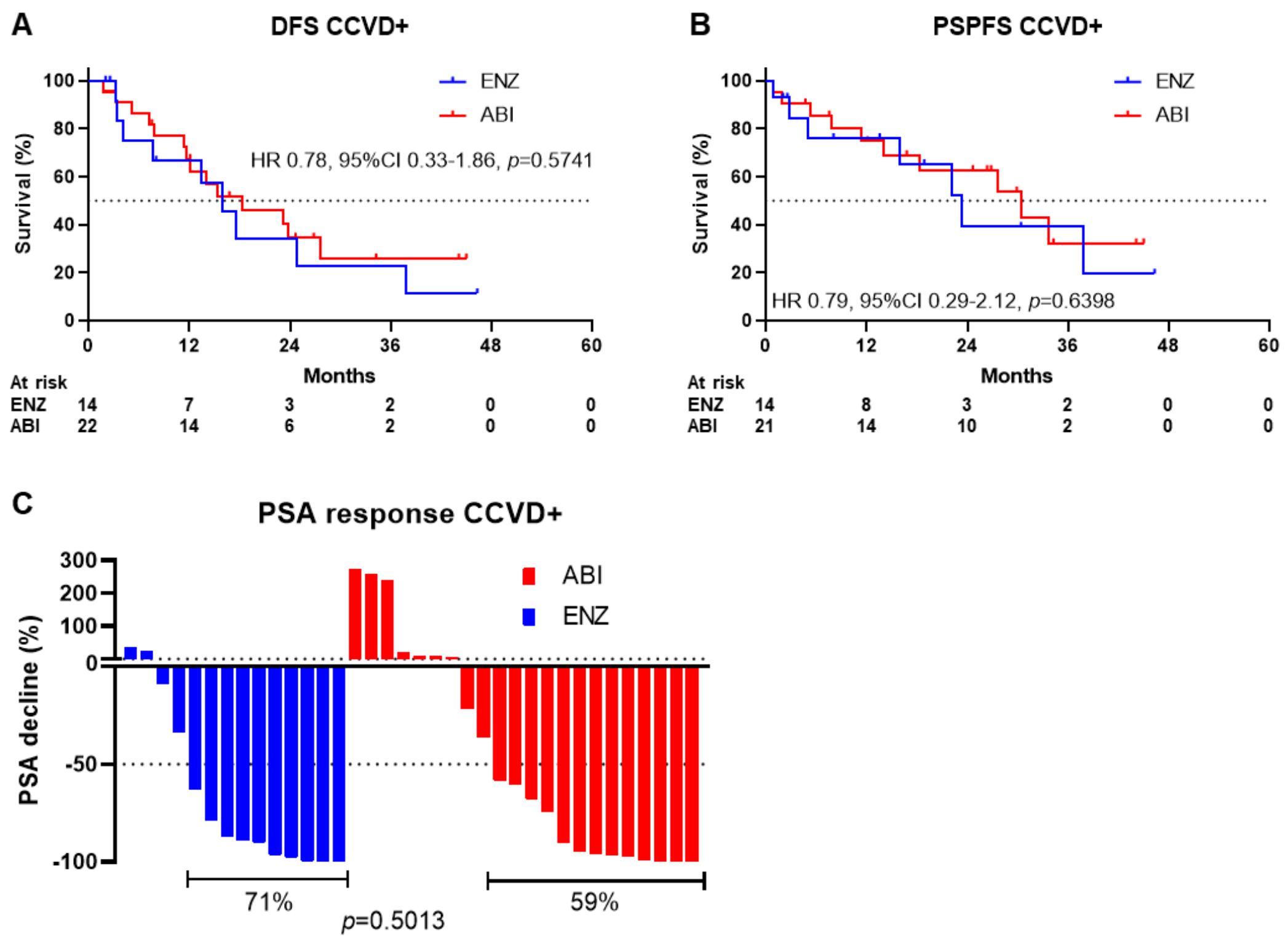

Supplement: Supplementary file 1 [file 2433-3298-9-5-1350-s001.pdf]
